# Comparison of Oral Preoperative Clonidine with Infusion of Intraoperative Labetalol on Bleeding during Tympanoplasty Surgery

**DOI:** 10.22038/ijorl.2025.78101.3626

**Published:** 2025

**Authors:** Mohamad Reza Afzalzadeh, Mostafa Mahdavi, Saleheh Asghari, Maryam Emadzadeh

**Affiliations:** 1 *Sinus and Surgical Endoscopic Research Center, Department of Otorhinolaryngology, School of Medicine, Mashhad University of Medical Sciences, Mashhad, Iran.*; 2 *School of Medicine, Mashhad University of Medical Sciences, Mashhad, Iran.*; 3 *Department of Anesthesiology and Critical Care, Dr. Ali Shariati Hospital, Tehran University of Medical Sciences, Tehran, Iran.*; 4 *Clinical Research Development Unit, Ghaem hospital, Mashhad University of Medical Sciences, Mashhad, Iran.*

**Keywords:** Tympanoplasty, Bleeding, Clonidine, Labetalol

## Abstract

**Introduction:**

Surgical bleeding is one of the most critical complications in various surgical procedures. In middle ear surgery, managing bleeding is a significant challenge for anesthesiologists because even minor bleeding can obstruct the surgeon's vision and prolong the surgery. Our objective is to compare the impact of preoperative oral clonidine versus labetalol infusion on bleeding volume during tympanoplasty surgery.

**Materials and Methods:**

In this double-blind randomized controlled trial, tympanoplasty candidates were randomly assigned to three groups: the clonidine group (received 300 micrograms of clonidine tablets one hour before surgery with normal saline infusion during the operation), the control group (given a placebo tablet one hour before surgery with normal saline infusion during surgery), and the labetalol group (administered a placebo tablet one hour before surgery with labetalol infusion at a rate of 0.2 mg/kg of body weight per hour during surgery). We then evaluated the extent of intraoperative bleeding, systolic and diastolic blood pressure, mean arterial pressure, and heart rate at various time points until the end of the surgery.

**Results:**

Clonidine and labetalol were effective in reducing intraoperative bleeding compared to the control group. Grade 2 bleeding (minimal bleeding requiring intermittent suction) was the highest grade observed across all three groups and was consistently noted in all patients. Clonidine demonstrated greater efficiency in reducing systolic and diastolic blood pressure, mean arterial pressure, and heart rate compared to both labetalol and the control group.

**Conclusion:**

Premedication with clonidine or labetalol is associated with reduced intraoperative bleeding, improved surgical field visibility, and shorter duration of tympanoplasty procedures. This may potentially lead to increased satisfaction and success rates of the operation.

## Introduction

Tympanoplasty surgery is a fundamental technique used for repairing the eardrum. The outcome and success of a tympanoplasty are contingent on the anesthetic techniques and bleeding control measures employed during the operation (1). Managing bleeding during middle ear surgery is a challenge for anesthesiologists because even minor bleeding can obstruct the surgeon's view and prolong the surgery (2). The act of severing blood vessels inevitably leads to bleeding; therefore, achieving meticulous hemostasis, while potentially challenging, is one of the fundamental principles of surgical practice (3). If the level of bleeding exceeds a certain threshold, it can lead to various complications and risks, including: a) Hemodynamic instability leading to reduced oxygen-carrying capacity, diminished blood pressure, decreased cardiac output, and impaired perfusion of vital organs (4); b) Detrimental effects within the surgical field, hindering the surgeon's view, extending the duration of the operation, and potentially causing harm to delicate structures surrounding the surgical site (5); c) Necessity for blood transfusions that encompasses the costs associated with blood collection, storage, and administration (5). Therefore, it is not surprising that in recent decades, efforts have been made to reduce intraoperative bleeding and implement strategies for preserving autologous blood.

Hemodynamic changes are significant risk factors for bleeding during surgeries, often due to sympathetic responses (6). Deliberate hypotension, an anesthetic technique, has been proven effective in reducing intraoperative bleeding and providing a clear operative field (7). Suggested techniques for inducing hypotension in middle ear surgeries include inhalation anesthetics, vasodilators, autonomic ganglion blockers, and adrenergic receptor antagonists (8). Controlled hypotensive anesthesia is commonly used to reduce blood loss in various surgeries. Administering preoperative medications that enhance the hypotensive effects of inhaled anesthetics, while avoiding the limitations of intravenous vasodilators, would be highly beneficial.

Clonidine is an antihypertensive agent that acts as a central alpha-2 adrenergic receptor, reducing sympathetic outflow. By stimulating central postsynaptic receptors and modulating noradrenaline release, clonidine exerts significant hypotensive effects and is well-established as an antihypertensive medication (9). Due to its influence on the sympathetic nervous system, particularly its ability to lower circulating epinephrine levels, clonidine is utilized across various medical fields. Moreover, it reduces anesthesia requirements and attenuates the reflexive cardiovascular responses associated with tracheal intubation (10). 

Labetalol is a combined α- and β- adrenergic receptor antagonist approved for both oral and intravenous administration in the management of hypertension. Labetalol's current approved indication includes the treatment of hypertension, either as a standalone therapy or in combination with other agents (11). 

In this study, we compared the efficacy of clonidine premedication with labetalol infusion to determine which one can result in the greatest improvement in intraoperative visibility by reducing bleeding, heart rate, systolic blood pressure (SBP), diastolic blood pressure (DBP), and mean arterial pressure (MAP) during tympanoplasty.

## Materials and Methods

In this double-blind, randomized clinical trial, patients were allocated into two intervention groups and one control group. The study was conducted at Ghaem Hospital in Mashhad, Iran. The Ethics Committee of Mashhad University of Medical Sciences approved the study protocol under approval number of IR.MUMS. MEDICAL.REC.1398.762. Moreover, its protocol was registered in the Iranian Registry of Clinical Trials (IRCT) (IR. MUMS. MEDICAL. REC.1398.762).

The inclusion criteria for patients were as follows: a) American Society of Anesthesiologists (ASA) grade 1 and 2, b) aged between 16 and 60 years old, c) eligible for tympanoplasty surgery, d) no history of previous systemic diseases, and e) had signed a written informed consent form. Exclusion criteria included pregnancy, breastfeeding, a history of heart conditions (bradycardia, heart block, SSS), liver, kidney, lung, or nervous diseases, as well as the use of beta-blockers and MAO inhibitors, allergic rhinitis, coagulopathy, local infections, and patients requiring mastoid bone surgery.

**Fig 1:**
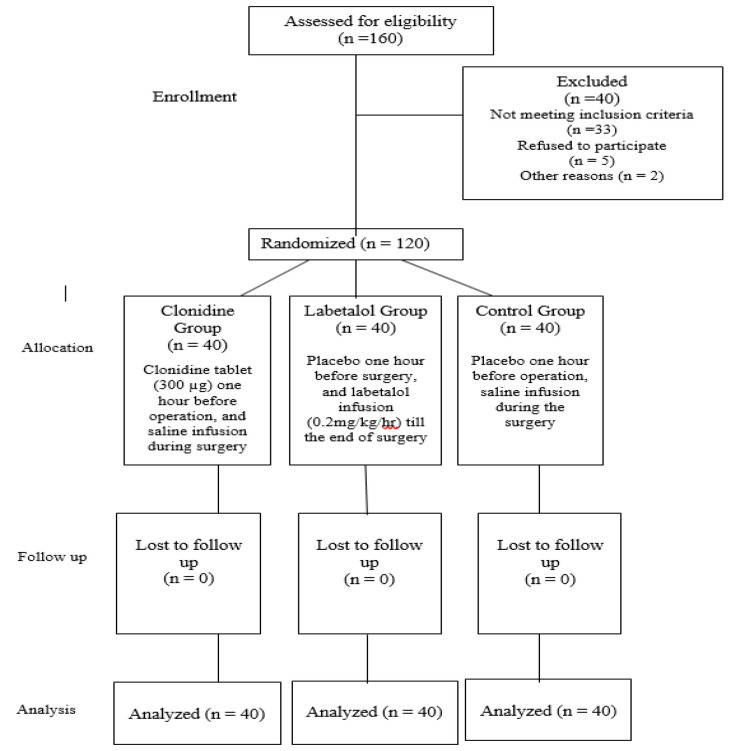
CONSORT diagram showing the participants through each group of the trial

Two intervention groups received hypotensive anesthesia, while the control group received normotensive anesthesia as follows: In group C, a *Clonidine* tablet (300 µg) was administered orally one hour before the operation, and saline infusion was done during the surgery. In group L, a placebo was used one hour before the surgery, and IV *labetalol* infusion was administered (0.2mg/kg/hr) until the end of the surgery. In the control group, a placebo was administered one hour before the operation with saline infusion during the surgery. All selected patients were fasted for at least eight hours before the surgery. Figure 1 shows the CONSORT diagram of the trial.

All patients were preloaded with oral ranitidine (20ml/kg) and lactated Ringer's solution before the surgery. Patients were monitored for blood pressure, electrocardiogram (ECG), heart rate, and SpO2. Preoxygenation with 100% O2 was conducted for 3 minutes. The induction and maintenance of anesthesia were consistent for all patients. Fentanyl was given to regulate increases in heart rate, while atropine was used if a decrease in heart rate was observed. Blood pressure was actively managed to stay within the range of 60-80 mmHg by adjusting the infusion rate of propofol. If there was a 20% decrease in blood pressure from the baseline, a Phenylephrine bolus was administered.

Intraoperative bleeding severity was assessed according to the following scoring system: 1) Minimum need for suctioning; 2) Slight bleeding, occasional suctioning required; 3) Slight bleeding, frequent suctioning required; 4) Bleeding threatens surgical field immediately after suction is removed; and 5) Uncontrolled bleeding. Patients who met the inclusion criteria were randomly allocated into three equal study groups: C (clonidine), L (labetalol), and a control group (N). This randomization was done using a computer random number generator. After randomization, the codes were written on separate papers placed in opaque envelopes. Each time a new eligible patient entered, the envelope was picked up and the patient received the medication indicated on the paper (C/L/N). Patients, anesthesia assistants, and the statistical analyzer were all blinded to the patients` randomization and data. The packaging of the drugs was similar to maintain blinding. Additionally, the nurse responsible for evaluating the outcomes was also blinded to the study groups. The primary outcome was defined as the severity of bleeding at the site of the surgery. Surgical field bleeding was assessed immediately following the elevation of the tympanomeatal flap (before manipulation of middle ear pathology). Secondary outcomes included SBP, DBP, MAP, and HR measured before anesthesia induction, before and after intubation, every three minutes for a total of thirty minutes, and subsequently every five minutes until the end of the surgery.Based on the percentage of bleeding in the study by Nederi et al. (12) in both intervention and control groups, and using G Power software, we calculated the required sample size of 16 individuals per group. Considering three study groups and a 20% dropout rate, each group consisted of 40 participants. To compare quantitative variables between the three independent groups, we used ANOVA or Kruskal-Wallis tests. The Chi-square test was used for categorical variables. The Friedman test was also performed to measure differences between time points. A p-value of <0.05 was considered statistically significant.

## Results

A total of 120 patients (55 males and 65 females) with a mean age of 36.57± 10.95 were included in the study. All patients who met the inclusion criteria, with ASA class 1 and 2, and were candidates for tympanoplasty were included. 

The groups did not have significant differences in terms of mean age and gender distribution. Demographic characteristics of the patients are summarized in Table 1.

**Table 1:** Demographic characteristics of the patients in each group

**Groups**	**Age (mean ± SD); median (IQR)**	**Gender (Female/male)**
Clonidine (C)	38.07±11.93; 37.5 (27.25,47.75)	23/17
Labetalol (L)	35±11.81; 35 (25.5, 47.75)	24/16
Control (N)	36.65±8.88; 36.5 (30.25, 44.5)	18/22
p-value	0.45*	0.35**

SD: standard deviation; IQR: interquartile range * Kruskal-Walli’s test **Chi square test

Table 2 describes the primary outcome in the study groups. According to this Table, the majority of patients had a bleeding intensity grade of 2 (50.8%), and 1.7% of the patients had a grade 5 bleeding intensity. Bleeding severity significantly differed between groups (p-value=0.016). The control group exhibited the highest bleeding intensity, while Group L and Group C were almost similar in terms of bleeding intensity (Table 2).

**Table 2:** Bleeding severity in each study group

**Grade**	**Clonidine group; n (%)**	**Labetalol group; n (%)**	**Control group; n (%)**
1	10 (25)	3 (7.5)	6 (15)
2	20 (50)	28 (70)	13 (32.5)
3	8 (20)	8 (20)	11 (27.5)
4	2 (5)	0 (0)	9 (22.5)
5	0 (0)	1 (2.5)	1 (2.5)

**Fig 2:**
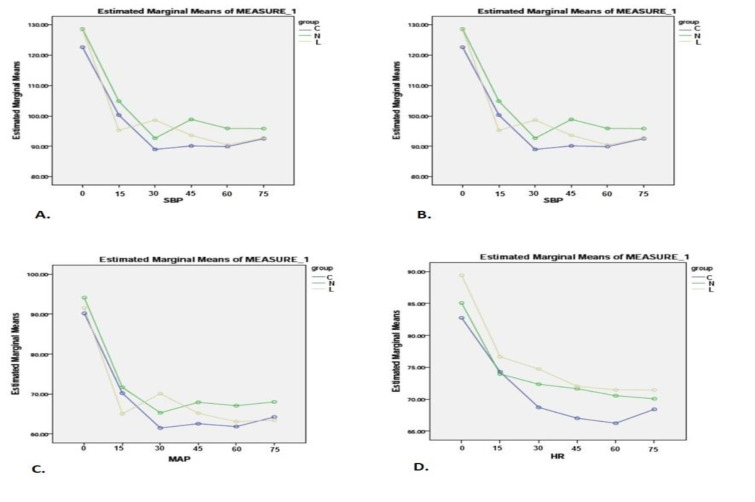
Systolic blood pressure (A), diastolic blood pressure (B), mean arterial pressure (C), and heart rate (D) changes at 15-minute intervals. Blue line: clonidine group, yellow line: labetalol group, green line: control group.

Table 3 and Figure 2 show the secondary outcomes at 15-minute intervals for each group.

There were no significant differences in baseline SBP and DBP between the groups (p-values: 0.47 and 0.74, respectively) (Table 3 and Figures 2A and 2B). Both SBP and DBP significantly decreased compared to baseline in each study group (p-value: <0.001). At 30 and 45 minutes of follow-up, both systolic and diastolic blood pressure were significantly lower in the Clonidine group, while at other time points, there were no significant differences between the groups.

All three groups experienced the most substantial reduction in DBP within the first 15 minutes of the operation. 

Table 3 and Figure 2 (C) show MAP changes at 15-minute intervals in each group. Although MAP was significantly decreased in each group compared to the baseline measurements, the control group exhibited the smallest changes in MAP after 80 minutes.

**Table 3:** Blood and mean arterial pressures in different time points in each study group

		**Clonidine group**	**Labetalol group**	**Control group**	**P-value ** ^b^
SBP	Baseline 15 min 30 min 45 min 60 min 80 min P-value ^a^	145.25±20.89 100.3±15.9 89±11.8 90.5±9 89.92±11.5 90.25±12.6 <0.001	144.9±21.19 95.25±21.27 98.6±15.3 93.6±17.5 90.45±13.28 92.9±11.04 <0.001	150.62±27.35 104.8±16.4 92.6±11.8 98.8±10 95.9±11.712 96.4±11.5 <0.001	0.47* 0.62** 0.006** 0.011** 0.057** 0.067*
DBP	Baseline 15 min 30 min 45 min 60 min 80 min P-value ^a^	86±12.3 52.6±14.8 44.62±9.9 45.6±9.6 46.3±9.9 47.9±9.8 <0.001	85.22±13.4 49.85±12.25 54.35±11.91 50.95±13.81 47.1±8.94 48.37±9.14 <0.001	87.95±21.2 55±12.4 48.9±10.3 51.6±9 50.2±9.1 51.42±10.5 <0.001	0.74* 0.21* <0.001** 0.041** 0.14* 0.23*
MAP	Baseline 15 min 30 min 45 min 60 min 80 min P-value ^a^	105.05±16.34 70.22±12.98 61.5±9.8 62.57±7.81 61.85±9.0763.02±10.25 <0.001	105.02±15.62 65.05±13.71 70.1±13.98 65.2±13.69 63.07±9.6 63.82±9.35 <0.001	106.5±25.07 71.67±11.13 65.3±8.61 67.92±7.13 67.07±8.49 67.55±9.87 0.001	0.92** 0.052* 0.003** 0.06** 0.03* 0.09**
HR	Baseline 15 min 30 min 45 min 60 min 80 min P-value ^a^	86.6±13.29 74.27±9.84 68.72±9.69 67.02±8.7 66.25±9.23 66.95±9.2 <0.001	87.7±21.92 76.65±19.35 74.72±14.97 72.02±14.36 71.47±13.88 70.47±12.63 <0.001	84.32±15.84 73.95±12.32 72.35±13.08 71.65±11.47 70.55±12 69.8±9.78 <0.001	0.67** 0.65** 0.11* 0.11** 0.11** 0.29**

SBP: Systolic blood pressure, DBP: diastolic blood pressure, MAP: mean arterial pressure, HR: heart rate. ^a ^Within group comparison was performed using Friedman test. ^b ^Between group comparison was performed using ANOVA (*) or Kruskal-Wallis (**) tests.

All three groups experienced the highest reduction in MAP within the first 15 minutes of the operation. After 60 minutes from the start of the surgery, MAP was fairly similar between groups C and L, but significantly lower when compared to the control group (p-value: 0.03). According to the results, clonidine was more effective than labetalol and placebo in reducing MAP between minutes 30 and 60. There were no significant differences in MAP during the final follow-up at the 80^th^ minutes of the surgery (p-value: 0.09).

Table 3 and Figure 2 (D) display the changes in HR at 15-minute intervals for each group. HR decreased significantly during the operation in all study groups. While group C had the lowest HR at minute 80, the difference between groups was not statistically significant. The groups exhibited the greatest reduction in HR during the initial 15-minute interval of the operation.

## Discussion

Blood-sparing techniques reduce the need for blood transfusions and associated blood products, as well as prevent the potential adverse effects of hemolytic and non-hemolytic transfusion reactions. To minimize blood loss during surgical procedures and maintain hemodynamic stability, the administration of α-agonist medications like clonidine and labetalol has proven to be an effective strategy (13).

Our study demonstrated that clonidine premedication and labetalol infusion effectively enhanced the hypotensive effects of propofol, decreased blood loss, and improved visibility during tympanoplasty. This resulted in clear operative fields and satisfactory operating conditions in both groups. In terms of systolic blood pressure (SBP), group L initially showed a more significant decrease, followed by a slight increase. On the other hand, group C maintained a decreasing trend for a longer period. Between minutes 30 and 45, there was a significant difference in SBP among all three groups. However, by the end of the surgery, no significant differences were observed between the groups. It is important to note that these differences were not clinically significant.

Bleeding scores of three to five were significantly higher in the control group compared to both the clonidine and labetalol groups, indicating that frequent suctioning was necessary. These findings are consistent with previous studies in various surgical fields. The effectiveness of clonidine in controlling bleeding and maintaining hemodynamic stability has been evaluated in middle ear surgery (10,12,14-17), laparoscopic cholecystectomy, cesarean section, endoscopic sinus surgery, otologic surgery, orthopedic surgery, and oromaxillofacial surgery (18-23).

According to these studies, clonidine effectively manages intraoperative hemorrhage by regulating central noradrenergic hyperactivity and reducing hemodynamic responses (18,19,23,24).

Elevated blood pressure and heart rate are hemodynamic variables that can increase the risk of bleeding in the operative field. This can lead to blood loss, decreased quality of the operative field, and poor surgical outcomes, which are common complications during middle ear surgeries. (7,8). Therefore, maintaining hemodynamic variables within the physiological range ensures a bloodless and clear operative field.

In the pediatric population undergoing orthognathic surgery, the administration of 5 micrograms per kilogram of clonidine helped to control hypotension. This resulted in reduced requirements for isoflurane, fentanyl, and labetalol. Clonidine also helped to mitigate changes in heart rate and blood pressure, and provided a faster recovery from the anesthesia (25). Preoperative administration of clonidine has been shown to decrease intraoperative bleeding and improve hemodynamics in addicted patients compared to non-addicted patients. Authors recommend it as a premedication to reduce intra-operative blood loss and the adverse effects of hemodynamic instability (24). Ebneshahidi et al. demonstrated improved hemodynamic stability in patients undergoing cesarean sections following both endotracheal intubation and extubation. However, the significance of using clonidine in high-risk patients warrants further investigation (18). 

Despite the studies mentioned, the exact process through which controlled hypotension decreases blood loss is not fully understood. Earlier research has indicated that clonidine may decrease blood loss in paranasal sinus and spine surgeries without inducing hypotension, suggesting that clonidine's impact on tissue blood flow and blood loss may be attributed to mechanisms other than reducing blood pressure (22,26).

Clonidine, an alpha-2 adrenergic receptor agonist, induces sedative and analgesic effects by activating central α-2 adrenergic receptors located at various sites within the central nervous system. Activation of medullary α -2 adrenergic receptors reduces sympathetic activity and enhances vagal activity, resulting in a reduction of hemodynamic responses to stress-inducing stimuli. Furthermore, the stimulation of presynaptic alpha-2 adrenergic receptors leads to a decrease in norepinephrine release at peripheral sympathetic nerve endings, resulting in reduced sympathetic activity. 

The exact mechanism responsible for clonidine's enhanced vasoconstriction remains uncertain. It is suggested that the vasoconstrictive action of clonidine may involve the activation of post-binding alpha-1 adrenergic receptors. Additionally, clonidine has been observed to interact with the α-2b subtype of α -2 adrenergic receptors in peripheral vascular smooth muscle, leading to vasoconstriction. Clonidine is known to be rapidly absorbed after oral administration. A study by Toivonen et al. revealed a significant correlation between clonidine plasma concentration and its antihypertensive effect, although they did not observe a correlation with heart rate (14,27). 

In a comparison between labetalol and the beta-1 adrenergic blocker esmolol, labetalol did not show superiority in reducing blood loss, maintaining MAP control, regulating heart rate, or improving surgical visibility in middle ear surgery (28). In another study, the labetalol group had slightly higher amounts of bleeding, while surgeon satisfaction with the quality of the operative field was slightly higher in the nitroglycerin group. However, no significant difference was found between the two groups in relation to these factors (25). Based on the results of the current trial, clonidine was more effective in reducing heart rate compared to the labetalol and control groups. Both labetalol and clonidine had similar effects on primary and secondary outcomes. Furthermore, their main effect in decreasing MAP, SBP, and DBP occurred within the first 15 minutes of the surgery. By evaluating the role of clonidine in infiltration block techniques during local anesthesia for middle ear surgery, it was found that 30 µg of clonidine provided better pain relief during the initial hours and prolonged the time of sensory analgesia. However, it had no effect on the onset of anesthesia or total analgesic consumption (10). Consistent with these findings, preanesthetic medication with clonidine has been shown to be beneficial in suppressing reflex tachycardia and hypertensive responses related to intubation in both adults and children (24,29). Matot et al. also suggested the routine use of clonidine for patients undergoing laryngoscopic or bronchoscopic procedures (30). None of the patients in our study experienced significant adverse effects following the administration of clonidine and labetalol such as uncontrolled hypotension and bradycardia. Comparing the groups revealed a similar requirement for fluid challenge, supporting the safety and efficacy of controlling bleeding and hemodynamic variables following premedication with clonidine or labetalol.

## Conclusion

Both α-agonist drugs used in the current study, clonidine and labetalol, were effective in maintaining hemodynamic stability during tympanoplasty, and neither was found to be superior to the other. These medications demonstrated minimal adverse effects when administered at appropriate doses and under careful monitoring. Moreover, their application led to a reduction in bleeding during tympanoplasty.
